# Lymphopenia: A useful predictor of COVID-19 disease severity and mortality

**DOI:** 10.12669/pjms.37.7.4085

**Published:** 2021

**Authors:** Kaleem Ullah Toori, Muhammad Arsalan Qureshi, Asma Chaudhry

**Affiliations:** 1 Dr. Dr. Kaleem Ullah Toori, FRCP (Glasgow). Department of Medicine, KRL Hospital, Islamabad, Pakistan; 2 Dr. Muhammad Arsalan Qureshi (M.B.B.S). Department of Medicine, KRL Hospital, Islamabad, Pakistan; 3 Dr. Asma Chaudhry, MRCP (UK), FCPS General Medicine (Pakistan). Department of Medicine and Endocrinology, Southend University Hospital, Southend-on-Sea, United Kingdom

**Keywords:** COVID-19 RT-PCR, Disease severity (asymptomatic, mild, moderate and severe), Lymphopenia

## Abstract

**Objective:**

To detect association of lymphopenia with disease severity and mortality.

**Methods:**

Total 874 COVID RT-PCR positive patients admitted to KRL Hospital Islamabad from April 2020 to August 2020 were included in this cross-sectional study. Lymphopenia was defined as <1100 cells/micro–L. WHO categories for disease severity were used. Demographic profile, absolute lymphocyte counts and co-morbidities were recorded. Pearson’s Chi Square test was used to see association between lymphopenia and disease severity as well as disease outcome. Regression analysis was used to see whether lymphopenia would predict disease severity. Comparison of means of absolute lymphocyte count in different disease categories was done by ANOVA. Tukey’s test range was then used to find the means different from each other. P-value ≤ 0.05 was considered statistically significant

**Results:**

The mean age of patients was 40 ± 12.3 years. Majority patients (73.9%) were asymptomatic. Lymphopenia was present in 6.9% of total patients. Significant association was found between lymphopenia and disease severity as well as lymphopenia and mortality (< 0.001). Lymphopenia was found to be a predictor of disease severity using regression analysis (< 0.001). Comparison of mean absolute lymphocyte count was significant among disease severity categories (< 0.001). On post-hoc analysis, difference in absolute lymphocyte count was significant moving from asymptomatic to mild and then moderate disease category. However no significant difference was seen in absolute lymphocyte count between moderate and severe categories.

**Conclusion:**

Results are compatible with worldwide studies and lymphopenia is valid as a marker of disease severity and mortality.

## INTRODUCTION

Corona virus disease (COVID-19) first emerged from Wuhan in China and spread rapidly to different parts of the world. It was declared a pandemic by the World Health Organization (WHO) on March 11, 2020.^1^ The virus has affected millions of people. As of 21^st^ June, 2021, there are 178, 118,597 confirmed cases of the virus and more than 3,864,180 deaths that have been reported to the WHO.^2^ Pakistan’s index cases was reported back in February 2020 in Karachi and Rawalpindi.^3^ The number of cases in the country rose to 948,268 with almost 22,000 deaths in June, 2021.^4^ As Mass vaccination drive have started throughout the country after approval of multiple Covid-19 vaccines, it can be expected that the course of the disease may change.

It has been postulated that the spread of disease was mainly by human-to-human contact.^5^ The signs and symptoms of the disease are not limited to a particular body system, rather a range of presentations occur, requiring isolation in asymptomatic or mild cases and oxygen, mechanical ventilation and ICU care with more severe presentation.^6^ The WHO has categorized the disease as asymptomatic, mild, moderate, severe and critical. Case positivity is defined by positive RT-PCR regardless of presence or absence of symptoms.^7^

Researchers have tried to come up with possible immunological parameters that might help to predict the severity of disease or a clinical outcome. Many hematological parameters are currently under interest to predict outcomes and mortality. Lymphocyte count has been of keen interest to clinicians all over the world probably as it is economical and easily available. Numerous studies all over the world^8^-^12^ have revealed that lymphopenia is an immunological finding in patients infected with corona virus and may be used as a prognostic marker. It is also a marker that predicts mortality amongst critically ill COVID-19 patients.

The aim of this study was to see whether a simple investigation like reduced lymphocyte count was associated with COVID-19 disease severity and mortality. We wanted to identify if lymphopenia could be used as a predictor of disease severity. We also wanted to see how the absolute lymphocyte count would vary amongst disease severity categories. Methods to achieve this are mentioned in detail below. Limited data is available regarding these parameters in the region as COVID-19 is still an emerging pandemic.

## METHODS

This was a hospital-based, descriptive cross-sectional study, conducted at KRL hospital Islamabad where a consecutive series of patients 1^st^ April 2020 to 31^st^ August 2020 were studied. Sample size calculation was not applicable as there is no data on prevalence of the disease in Pakistani population or internationally.^13^ The study was started after taking approval from the hospital’s ethical and research committee (Ref ERC: KRL-HI-ERC/Dec20/27, dated March 30, 2020). All patients were employees of the KRL organization. Our organization developed an isolation facility for the COVID-19 positive patients. Written informed consent was taken from all patients.

Patients who presented with possible signs and symptoms of COVID-19 were first isolated and Reverse transcription polymerase chain reaction (RT-PCR) of nasopharyngeal swabs was performed. Those with a positive RT-PCR were admitted and included in the study. History, demographic profile, exposure, drug history, comorbidities and relevant medical details were obtained and recorded. Investigations including blood tests and radiology were performed as per hospital’s protocol. All X-rays and CT scans were reported by a classified radiologist. Lymphopenia was calculated using Complete Blood Count with differential count. The definition of Lymphopenia adapted is <1100 cells/micro-L.^8^ Lymphopenia was documented on day of admission and then serial testing during stay at the hospital.

Case severity definitions were as per the interim guidance of WHO.^7^ Asymptomatic (patients are RT-PCR positive but do not show symptoms), mild (patients are RT-PCR positive but no hypoxia), moderate (RT-PCR positive patients who show signs of pneumonia and but no signs of severe hypoxia with Spo2 > 90%), severe (signs of severe pneumonia evident with respiratory rate more than 30 breaths/minute or Spo2 < 90% and critical (with Acute Respiratory Distress Syndrome [ARDS] or septic shock). We included both severe and critical patients under category of severe.

### Statistical analysis

Statistical analysis was performed using IBM SPSS 25. Continuous variables like age were expressed as mean values with standard deviation. Frequency and percentages were calculated for categorical variables like cases as per severity, symptoms, comorbidities and lymphopenia. Categorical variables were tested using Pearson’s Chi square test to check for statistical significance. Comparison of mean values was done by one way Anova test and Tukey’s range test was used to find the means different from each other. Simple regression analysis was applied to determine whether lymphopenia would be a predictor of disease severity. P-value ≤ 0.05 was considered statistically significant. Confidence interval was at 95%.

## RESULTS

Total 874 patients were analysed during this study. The mean age of patients was 40 ± 12.3 years (range 12 - 87 years). Majority were males i.e., 96% with only 4% being female. Out of the total patients admitted, 73.9% (n=646) were asymptomatic while 26.1% (n=228) were symptomatic. Among the symptomatic, the most frequent symptoms were fever 87.3% (n=199), cough 54.8% (n=125), dyspnoea 32% (n=73), myalgia 28.5% (n=65), sore threat 16.7% (n=38), diarrhoea and vomiting 5.2% (n=12) headache 3.1% (n=7), rhinorrhoea 2.2% (n=5) and haemoptysis 0.41% (n=1), in order of decreasing frequency.

On reviewing co-morbidities, 84% (n=734) of patients had no pre-morbidity while 16% (n=140) had underlying chronic medical conditions. The reported pre-morbid conditions were diabetes mellitus in 8.7% (n=76), hypertension in 8.2% (n=72), ischemic heart disease in 2.9% (n=25), chronic respiratory disease in 2.5% (n=22), chronic kidney disease in 1.7% (n=15), chronic liver disease in 0.5% (n=4), chronic neuro-psychiatric condition in 0.2% (n=2) and malignancy 0.1% (n=1)

Regarding disease severity, 73.9% (n=646) were asymptomatic, 16.7% (n=146) had mild disease, 4.5% (n=39) had moderate disease and 4.9% (n=43) had severe disease. 98.2% (N=858) of patients recovered while 1.8% (n=16) of patients died.

Lymphopenia was present in 6.9% (n=60) of total patients. Association between disease severity and lymphopenia is reported in Table-I. Lymphopenia was more frequent in moderate and severe disease, which was significant by applying Pearson Chi square proving a positive association of lymphopenia with disease severity. Using regression analysis we also found that lymphopenia was a significant predictor of disease severity, the results are reported in Table-I.

**Table I:** Association between disease severity and lymphopenia- Pearson Chi square and Regression Analysis.

*Disease Severity Categories*	*Lymphopenia n (%)*	*Pearson’s χ^2^ value ( p ) [d.o.f]*	*Regression*
Asymptomatic	19 (2.9%)	183.36 (< 0.001) [3]	R^2^= 0.17, F(1,869 ) = 186.48, p = (< 0.001)
Mild	7 (4.8%)		
Moderate	12 (30.8%)		
Severe	22 (51.2%)		

d.o.f: Degree of freedom.

Further analysis was done to compare the means of absolute lymphocyte count amongst disease severity categories using ANOVA. The comparison of means was significant as reported in Table-II. We then applied the Tukey’s range test to find the means that were significantly different from each other. The difference in mean lymphocyte count is statistically significant from asymptomatic to mild, and mild to moderate categories. However no significant difference was noted in moderate to severe category as reported in Table-III.

**Table II:** Variability of absolute lymphocyte count across all categories of disease severity- ANOVA.

		*Disease Severity*			

		*Asymptomatic*	*Mild*	*Moderate*	*Severe*

	*Mean (95% CI)*	*2542.5 (2465, 2620)*	*2143.5 (2004, 2282)*	*1471.3 (1190, 1752)*	*1121.2 (989, 1253)*
Absolute Lymphocyte Count	Minimum	572	744	408	510
	Maximum	6888	4074	4448	2340
	SD	39.4	70	138	65
	N	551	96	33	40

CI: Confidence interval, SD: Standard Deviation, N: Number of individuals.

**Table III:** Difference in mean absolute lymphocyte range amongst disease severity- Tukey’s range test

	*Mean Difference*	*Standard Error*	*p-value*	*Lower bound*	*Upper bound*
Asymptomatic- Mild	398.97*	96.28	<0.001	151.03	646.92
Mild-Moderate	672.22*	175.68	0.001	219.83	1124.62
Moderate-Severe	350.08	204.73	0.319	-177.13	877.30

Among the patients (n=16) who died of COVID-19, majority (56.3%) were found to have lymphopenia. The recovered group had fewer cases of lymphopenia. Pearson Chi square test was used to test the association between mortality and lymphopenia, reported in Table-IV. This reveals a significant association between mortality and lymphopenia.

**Table IV:** Frequency between lymphopenia and mortality- Pearson Chi square.

*Outcome*	*Lymphopenia n (%)*	*Pearson’s χ^2^ value (p) [d.o.f]*
Recovered	51 (5.9%)	62.17 (< 0.001) [1]
Died	9 (56.3%)	

d.o.f: degree of freedom.

## DISCUSSION

COVID-19 is an emerging pandemic which has caused havoc around the world. A lot of investigations are ongoing to identify disease markers of severity and mortality. Lymphocyte count is one of the haematological markers under investigation. Lymphopenia occurs due to a variety of reasons in COVID-19 patients. Cytokine storm is a phenomenon of excessive inflammatory reaction and has been associated with lymphopenia, with greater levels of inflammatory markers (TNF alpha and Interleukin 6 [IL-6]) causing more lymphocyte death. Mortality in COVID-19 patients was also linked with lower lymphocyte count in American studies.^10^ According to Mazzoni et al.^11^, high IL-6 levels lead to impaired immune cytotoxic activity of lymphocytes which are necessary to fight against viral illnesses. COVID-19 may also result in T cell exhaustion via increased expression of certain cell surface proteins with one Chinese study concluding that markers of T cell exhaustion are over expressed in COVID-19 patients in the ICU.^12^

The mean age of patients developing COVID-19 is variable. The mean age in our population was 40 years which is lower as compared to other Pakistani studies.^14^,^15^ Our patients were predominantly males which is keeping with other studies as COVID-19 is known to have a predilection for male gender.^14^,^15^

Majority patients in our study were asymptomatic. This is similar to data acquired by WHO which suggests up to eighty percent patients maybe asymptomatic.^16^ Amongst the symptomatic individuals, the most common clinical symptoms were fever, respiratory symptoms, and myalgias which are similar to those described by the WHO interim guidance.^7^

The study at our center has shown significant results with regards to relationship of lymphopenia and disease severity. Moderate and severe cases had statistically significant lymphopenia. We also found that as the disease severity increases from asymptomatic to mild and then moderate disease, the absolute lymphocyte count falls steadily and significantly. As disease progressed from moderate to severe, there was a fall in absolute lymphocyte counts, however this was not significant. We attributed this non-significance to very small number of patients in both moderate (4.5%) and severe (4.9%) categories. This difference might be better evident if greater numbers of moderate and severe patients would be included in subsequent studies. Lymphopenia in COVID-19 has been a well-documented finding in international studies.^10^-^12^ The results of our study are seconded by a Korean study which concluded that lymphopenia was more prominent in severe cases as compared to mild cases.^17^ Pakistani studies also documented significant lymphopenia in Intensive Care Unit patients.^14^,^15^

We also established a link between lymphopenia and mortality associated with COVID-19 patients. Meta-analysis of 23 studies involving more than 3000 patients also confirmed that greater degree of lymphopenia is associated with poor clinical outcome and that those who died had lower lymphocyte counts which was statistically significant; further endorsing what our study has shown.^8^

The results of our study are consistent with the above-mentioned studies which supports the hypothesis that lymphopenia is an important indicator and degree of lymphopenia can be used as a marker of disease severity. Our study further adds to the evidence that lymphopenia correlates with severe and deteriorating COVID-19 infection in the Pakistani population. It can provide an objective sign to identify patients at risk of deterioration early, leading to improved management of critical patients.

### Limitations of the study

The major limitations in our study were the predominant male number patients as they were employees of the organization and were required to be admitted if they were positive for RT-PCR -2. On the other hand, female patients opted to isolate at home unless they had moderate to severe disease requiring hospital management. Secondly, this was a single-center study.

## CONCLULSION

The lymphocyte count is a simple, effective and cheap guide to predict disease severity and mortality outcome amongst COVID-19 patients. This is especially important in our country, where lack of resources may limit expensive investigations. Earlier detection of lymphopenia should alert physicians to monitor for deterioration and investigate appropriately.

### Authors` Contribution:

**KT** conceived, designed and did statistical analysis along with review of manuscript and accountable for the accuracy of the study.

**MAQ** contributed to data collection and manuscript writing.

**AC** did manuscript writing with final review of manuscript.
